# Functionalized Contact Lenses for Ocular Health: Detection and Treatment of Ocular Infections with Moxifloxacin-Loaded Cellulose Nanofibers

**DOI:** 10.1021/acsomega.6c02807

**Published:** 2026-06-10

**Authors:** Zekeriya Cetinkaya, Cemile Yilmaz, Sadik Kucukgunay, Ismail Ocsoy, Mustafa Nisari

**Affiliations:** † Department of Ophthalmology, Kayseri State Hospital, Kayseri 38010, Turkey; ‡ Department of Analytical Chemistry, Faculty of Pharmacy, 52958 Erciyes University, Kayseri 38039, Turkey; § Department of Medical Pharmacology, Faculty of Medicine, Kırşehir Ahi Evran University, Kırşehir 40100, Turkey; ∥ Faculty of Dentistry, Department of Medical Biochemistry, University of Nuh Naci Yazgan, Kayseri 38170, Turkey

## Abstract

Ocular bacterial infections present significant treatment challenges due to the tear pH and limited retention of topical ophthalmic medications. In this study, we engineered dual-functional contact lenses (fCLs) by integrating moxifloxacin (MFX)-loaded cellulose nanofibers (CNFs) with anthocyanin-based natural pH indicators. The incorporation of CNFs was designed to enhance surface hydrophilicity and facilitate MFX binding, while anthocyanin functionalization provided a visible colorimetric response to infection-associated pH changes. The structural and surface properties were characterized via UV–visible spectroscopy and scanning electron microscopy (SEM). Antibacterial and antibiofilm assays against *Escherichia coli* (*E. coli*), *Staphylococcus aureus* (*S. aureus*), and *Enterococcus faecalis* (*E. faecalis*) revealed that the CNF-integrated lenses exhibited marked biofilm inhibition against *E. coli* compared to nonnanofiber controls. Furthermore, the fCLs maintained functional stability within physiological pH values (6.5–7.4). These findings demonstrate that the developed fCLs offer a promising approach to the simultaneous monitoring and management of ocular infections.

## Graphical abstract

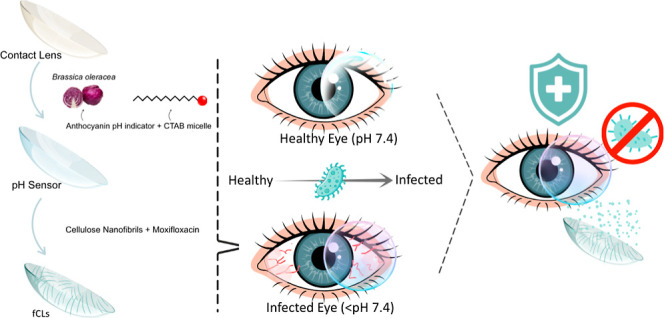

## 1 Introduction

Functionalized contact lenses (fCLs) have emerged as advanced platforms for the continuous monitoring of tear biomarkers and the comprehensive assessment of ocular health.^1^ By exploiting the tear-blood barrier, which allows physiological markers to diffuse into tear fluid, these wearable devices enable the noninvasive detection of systemic and ocular conditions, including diabetes and glaucoma, through the analysis of glucose levels and intraocular pressure.^2–5^ Advanced wearable sensors integrated with drug delivery systems present dual-function platforms that enable the simultaneous monitoring and treatment of eye diseases.^6^ Anthocyanin-loaded contact lenses have recently emerged as promising tools for visualizing physiological changes, specifically by detecting pH variations and biomarkers in tear fluids.^7–9^

Ocular bacterial infections are a significant public health problem, often causing severe complications, including corneal opacification and vision loss. Among these, bacterial keratitis is especially prevalent and poses a critical risk to ocular integrity.^10–12^ These infections can worsen the progression of the disease by establishing an acidic environment in the affected tissues.^13,14^ The monitoring of localized pH fluctuations in tear fluid provides a valuable diagnostic evaluation about the infection conditions.^15^ Although broad-spectrum topical antibiotics are the most common treatment strategy, conventional formulations exhibit intrinsic pharmacokinetics limitations such as low bioavailability, rapid precorneal clearance, and necessity strategies for frequent dosing. Consequently, there is a need for therapeutic strategies that enhance efficacy and retention while enabling early diagnosis and potential for improved infection control.

Cellulose-based materials represent an important group for drug delivery applications due to their inherent biocompatibility, viscoelastic properties, and tunable surface chemistry.^16–20^ Specifically, cellulose nanofibers (CNFs) can be easily functionalized owing to their reactive groups. While high water retention capacity of CNFs makes them ideal for ophthalmic applications, their porous structure effectively increases drug loading capacity.^21–24^ Previous studies have demonstrated that moxifloxacin (MFX)-loaded CNFs can disrupt the biofilm matrix of *Staphylococcus aureus* (*S. aureus*), consequently enhancing treatment efficacy through improved drug penetration.^25,26^ Siripongpreda and colleagues (2023) reported the potential of integrating commercial cellulose nanofibrils/levofloxacin/pH indicator dyes on the surface of contact lenses for diagnosis and treatment for ocular bacterial infection.^27^ Although organic indicators called “pH-responsive dyes”, like phenol red, methyl red, thymol blue, bromothymol blue, and phenolphthalein, have been used for the colorimetric detection of bacterial growth, toxicity and cost are essential drawbacks for their use of organic pH indicators, necessitating the development of safer alternatives.^27–29^

Herein, we report the development of dual-fCLs designed for enhanced antibiofilm efficacy and safe infection management. By integrating MFX-loaded CNFs with anthocyanin—a nontoxic, plant-derived natural pH indicator. We have generated a dual-function platform to overcome the drawbacks of conventional ocular delivery systems. These fCLs provide continuous antibacterial protection and simultaneous, rapid, colorimetric diagnosis of bacterial infections by using anthocyanin molecules to improve patient care in ophthalmology. Anthocyanins, known as “natural pH indicators”, are cost-effective, readily available, biocompatible agents. Additionally, these molecules exhibit distinct color changes owing to the protonation and deprotonation of hydroxyl groups in their structure.^30–34^ Consequently, the developed fCLs can be considered dual-functional wearable devices with potential for monitoring pH changes and supporting the treatment of ocular infections.

In contrast to previously reported contact lens-based theranostic systems, which largely rely on synthetic pH-responsive dyes and conventional drug loading approaches, this study introduces a biocompatible and naturally derived alternative by utilizing anthocyanin as a nontoxic pH indicator. Furthermore, the incorporation of cellulose nanofibers (CNFs) enhances the drug-loading capacity and enables controlled release behavior through intermolecular interactions with MFX. This dual-functional design provides a safer and cost-effective sensing platform while improving the antibiofilm performance. Collectively, these features distinguish the present system from existing approaches and highlight its potential as an integrated platform for the simultaneous monitoring and treatment of ocular bacterial infections.

## 2 Experimental Section

### 2.1 Materials and Instrument

AIR OPTIX plus HydraGlyde contact lenses (lotrafilcon B) (−1.00) were purchased from Alcon Laboratories Inc. (USA). MFX (≥98.0%, high-performance liquid chromatography) was obtained from Deva Holding (Turkey). Cetryltrimethylammonium bromide (CTAB, ≥99.9%), sodium dihydrogen phosphate monohydrate (NaH_2_PO_4_·H_2_O), sodium phosphate dibasic (Na_2_HPO_4_), sodium hydroxide (NaOH), hydrochloric acid (HCl), and ethanol (96.0%) were purchased from Merck and Sigma-Aldrich. Cellulose nanofibers (CNFs) were obtained by the Analytical Chemistry Research Laboratory, Faculty of Pharmacy, Erciyes University. All chemicals were of analytical grade and used without further purification. Red cabbage (*Brassica oleracea*) was purchased from a local organic market in Turkey. Bacterial strains *Escherichia coli* ATCC 25922 (*E. coli*), *Staphylococcus aureus* ATCC 25923 (*S. aureus*), and *Enterococcus faecalis* ATCC 29212 (*E. faecalis*) were obtained from the culture collection of the Department of Pharmaceutical Microbiology Research Laboratory, Erciyes University.

### 2.2 Preparation of Anthocyanins from Red Cabbage

Red cabbage (*Brassica oleracea*) was purchased from a local market in Kayseri, Turkey. The leaves were washed with distilled water and cut into small pieces. Subsequently, 100 g of the leaves were added to 100 mL of distilled water and boiled for 30 min (min). After cooling, the mixture was filtered using Whatman No. 1 filter paper. The extract was stored at −20 °C.^28,35^

### 2.3 Preparation of fCLs

The preservatives and surface-active components on the surface of the commercial contact lenses were washed and removed from polymeric structures. For this purpose, the lenses placed in a beaker containing 2 mL of distilled water were stirred for 24 h (hrs) at room temperature (RT: 25 ± 2 °C) under gentle magnetic stirring. The functionalization process is shown in Scheme 1. First, a micellar solution was prepared by dissolving 25% (w/w) anthocyanin extract and 0.1% (w/v) CTAB in a 50% (v/v) ethanol–water mixture under stirring for 30 min. MFX was incorporated into the CNF suspension to obtain final concentrations of 0.1% (w/v) CNF and 1 mg/mL MFX. This mixture was ultrasonicated for 30 min to ensure homogeneity. In the fCLs, the pretreated lenses were immersed in vials containing 2 mL of the prepared solutions. The samples were incubated at +4 °C for 7 days to allow maximum drug loading. Finally, the drug-loaded lenses were washed with distilled water to remove excess particles prior to characterizations.

**Scheme 1:**
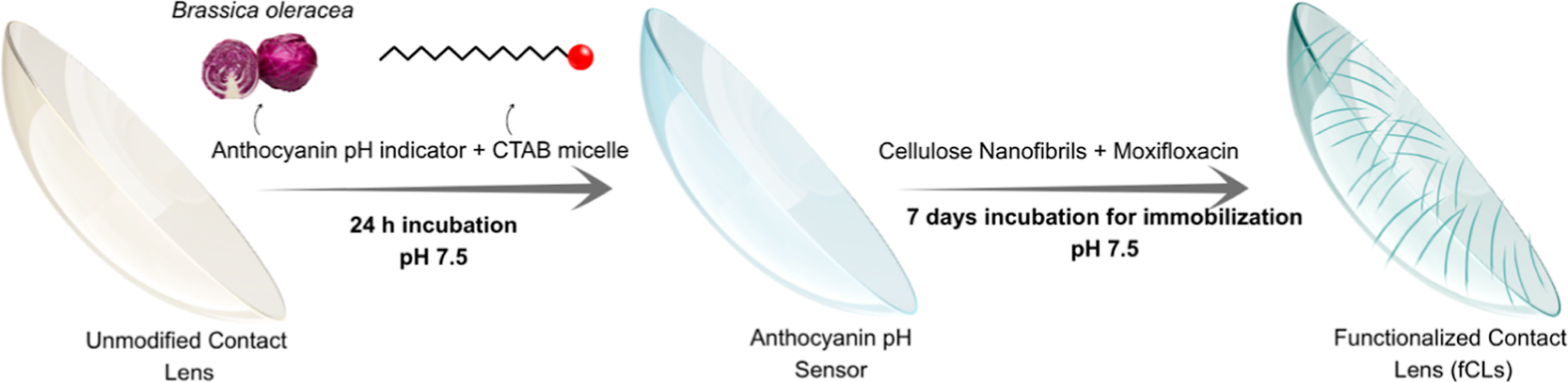
Schematic Illustration of Unmodified Contact Lenses Versus Functionalized Contact Lenses Incorporating a Natural pH Indicator and CNF/MFX (Created with https://www.canva.com/)

### 2.4 Characterization of fCLs

The surface morphologies of the fCLs were observed by scanning electron microscopy (SEM, ZEISS, Germany). The characteristic absorbance spectra of anthocyanin, MFX, and nanocomposite loaded onto lenses were analyzed using a UV–visible spectrophotometer (UV-1900i, Shimadzu, Japan). Surface chemical compositions were analyzed by Fourier transform infrared spectroscopy (FTIR, PerkinElmer Spotlight 400, USA). The fCLs were specifically characterized at pH 7.5, mimicking tear pH. To ensure that the surface pH during FTIR analysis was equivalent to physiological conditions, fCLs were equilibrated in simulated tear fluid (pH 7.5).

### 2.5 Drug Release Experiment

The release profiles of MFX from the fCLs and MFX-loaded contact lenses were evaluated under sink conditions to simulate the ocular environment. The drug-loaded lenses were immersed in solutions containing (2 mL) phosphate-buffer saline (PBS, 0.01 M, pH 7.4). At predetermined different time intervals (0.5, 1, 2, 3, 4, 5, 6, and 24 h), 1 mL of the release medium was immediately replaced with an equal volume of fresh PBS to maintain a constant volume and sink conditions. The concentration of released MFX in the samples was measured by measuring the absorbance at λ_max_ (290 nm) using a UV–visible spectrophotometer (UV-1900i, Shimadzu).

### 2.6 Antibacterial and Antibiofilm Activities of fCLs

Time-dependent release of MFX from the surface of fCLs, including CNF/MFX and MFX, at different pH values (pHs) was evaluated against pathogenic strains. The release rates were analyzed in terms of antibacterial activity and rates against *E. coli*, *S. aureus*, and *E. faecalis* strains. The bacterial strains were cultured for 24 h at 37 °C. The cell densities of the obtained bacterial strains were adjusted to 1 × 10^8^ CFU/mL. Subsequently, 10 μL of each strain was added into the wells of a 96-well microplate with Mueller-Hinton broth. Plates were incubated for 24 h at 37 °C. For bacterial inhibition rates, plates were analyzed by spectrophotometric analysis with an ELISA system (Ao Microplate Reader, Azure Biosystems, Inc.). The experiments were carried out in triplicate. The killing efficiency of the fCLs was assessed using a previously reported formula.^36^

Contact lens surfaces are prone to biofilm formation by pathogenic bacteria.^37^ *E. coli* was cultured in tryptic soy agar broth at 37 °C. Isolated *E. coli* colonies were transferred to brain-heart infusion (BHI) medium. The unmodified contact lenses and the fCLs, including CNF/MFX composites, were placed separately in a 24-well plate. The wells were filled with 1% glucose, 10 μL of *E. coli* culture (adjusted to 0.5 McFarland standard), and 1 mL of BHI medium. Biofilm formation was allowed to proceed for 72 h of incubation, with lenses being transferred to fresh medium every 24 h. Finally, biofilm formation on the lens surfaces was characterized by SEM.

## 3 Results and Discussion

The unmodified contact lens, the contact lens including only anthocyanin, and the fCLs including anthocyanin and CNF/MFX composites were monitored at different pHs.

The commercial contact lens was rinsed with distilled water to remove any salts and dried at 25 ± 2 °C before surface modification. The clean, unmodified contact lens, shown in Figure 1A, appeared transparent. After soaking it in a mixture of pH indicator and CTAB, the color of the anthocyanin-stained contact lens became purple between pH 5 and 7 in Figure 1B. The color of the lens turned into a light blue and green at pH 7.5 and pH 8, respectively, as shown in Figure 1B. The color intensity at the edges of the lens was greater than at the center due to the increased thickness of the anthocyanin at the edges. For the production of the fCLs lens, the prepared pH-responsive anthocyanin/CTAB-loaded contact lens was immersed in the mixture CNF/MFX nanocomposite. The color changes on the fCLs were consistent with the results shown in Figure 1B,C. A slight color change was observed, but no agglomeration of the CNF/MFX nanocomposite on the lens surface was observed as shown in Figure 1C.

**Figure 1:**
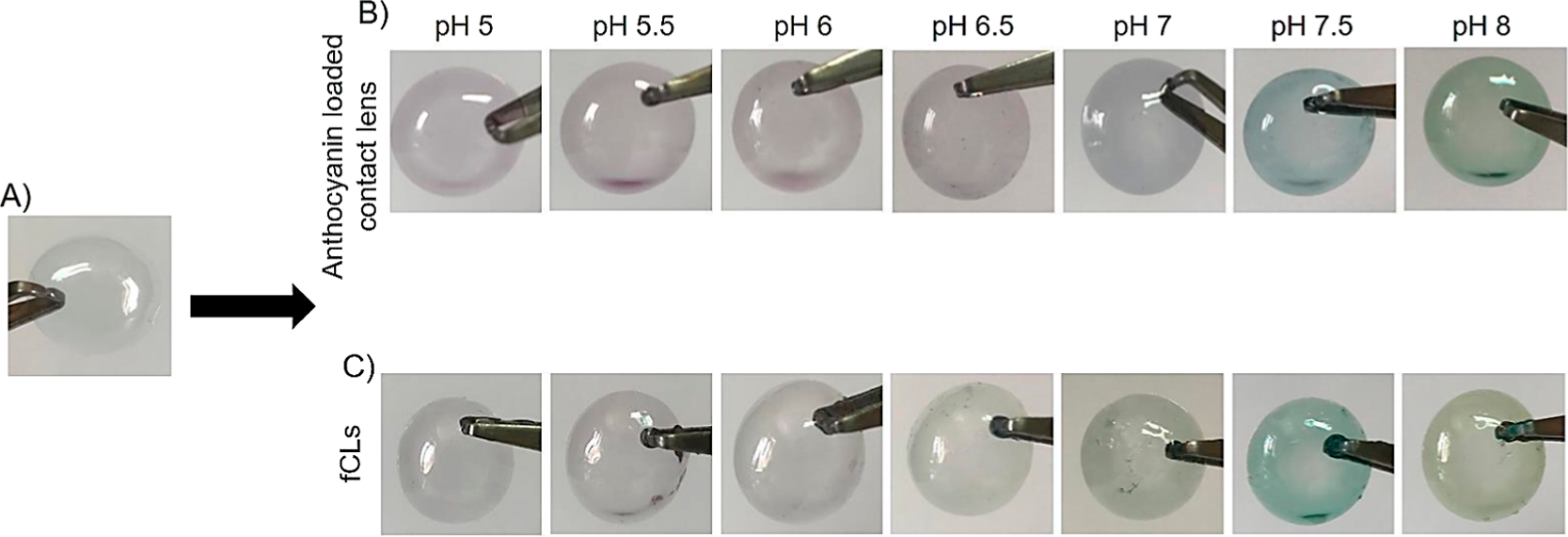
Appearance of (A) unmodified, (B) anthocyanin-loaded contact lenses, and (C) functional contact lenses (fCLs) at different pH levels (pH 5–8).

The unmodified contact lens has a transparent color and maintains its natural transparent color at the different pHs (Figure 2A). Both the anthocyanin-stained contact lens and fCLs showed various colors at the different pHs, owing to protonation and deprotonation of anthocyanin molecules, as seen in Figure 2B,C. While the color of both lenses was purple at pH 6.5, the anthocyanin molecules were deprotonated at pH 7–7.5 and pH 8, and then the colors of the lenses became a light blue and green, respectively (Figure 2B,C). When the pH of the reaction environment increases, the more alkaline pH leads to deprotonation of anthocyanin, and then, the corresponding colors are obtained.

**Figure 2:**
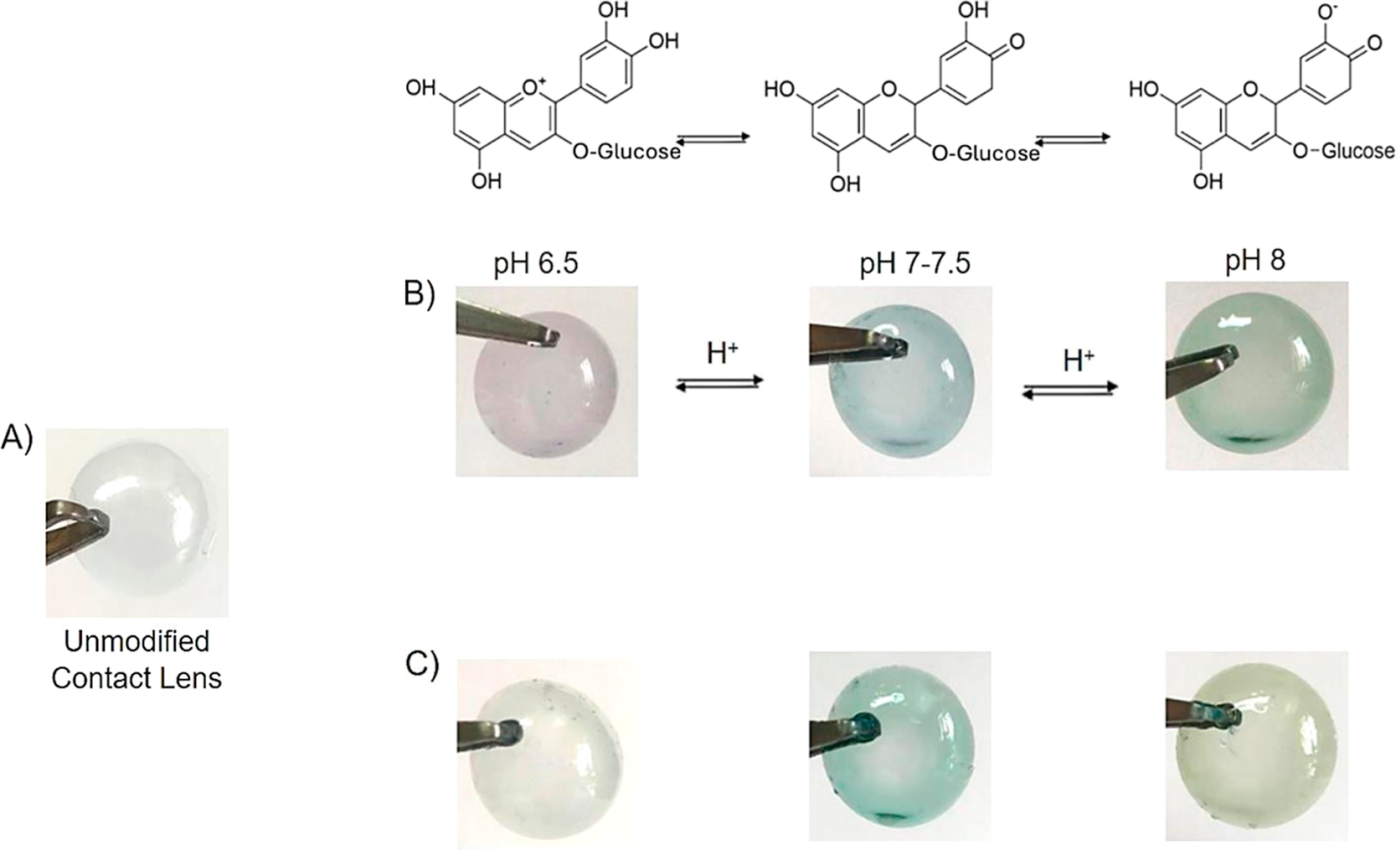
Photographs of (A) unmodified contact lens, (B) anthocyanin-stained contact lens, and (C) fCLs at the ideal ocular pHs.

The absorbance spectra of MFX and CNF/MFX nanocomposite solutions were obtained (Figure 3A), and the characteristic absorbance peaks of MFX itself and in the CNF/MFX nanocomposite were recorded at 290 and 339 nm. The absorbance spectra of the fCLs at different pH values were measured (Figure 3B). It is confirmed that MFX loading efficiency is much higher on the surface of the fCLs at pH 7–7.5 and pH 8 than at pH 6.5.

**Figure 3:**
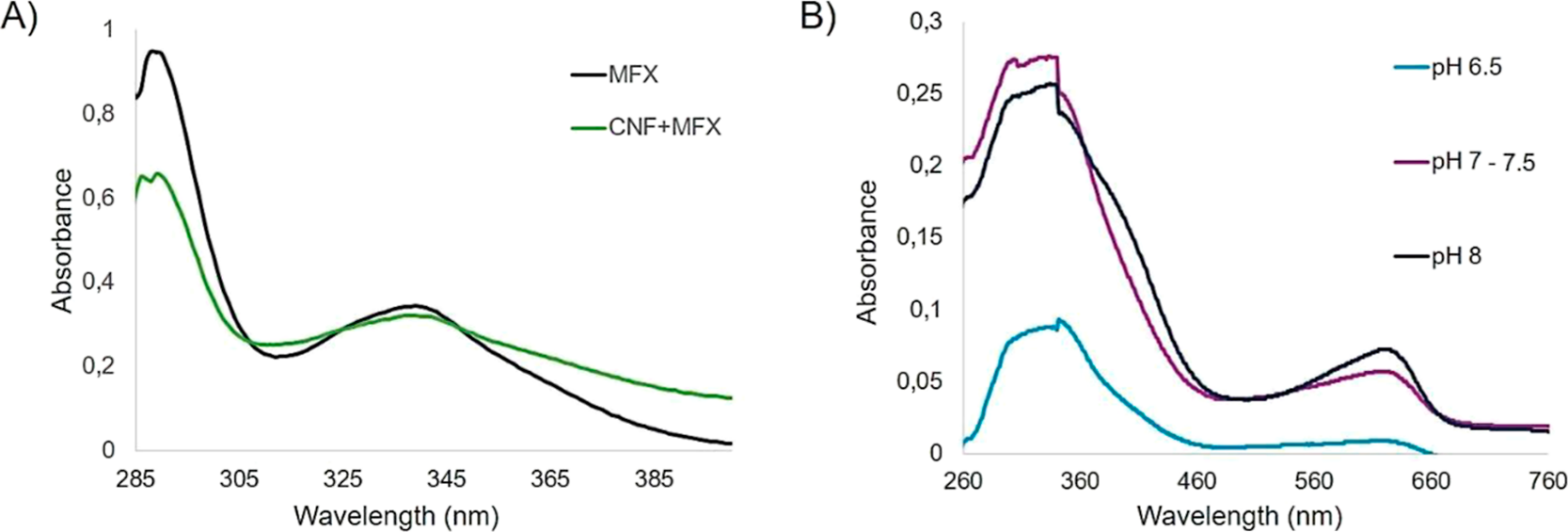
UV–visible spectrum of (A) MFX and CNF/MFX nanocomposite solutions and (B) fCLs at different pHs.

SEM images of contact lenses were obtained before and after modification to show differences on the surface of the contact lenses. The SEM image of the pristine contact lens called “unmodified” showed a relatively smooth and clean surface (Figure 4A). After modification with anthocyanin and CTAB, the SEM image of anthocyanin-stained contact lens shows the assembly of small particles on the surface of the contact lens (Figure 4B). Figure 4C demonstrated a crumpled structure on the surface of the fCL’s surface due to the presence of a CNF/MFX nanocomposite loaded on their surface.

**Figure 4:**
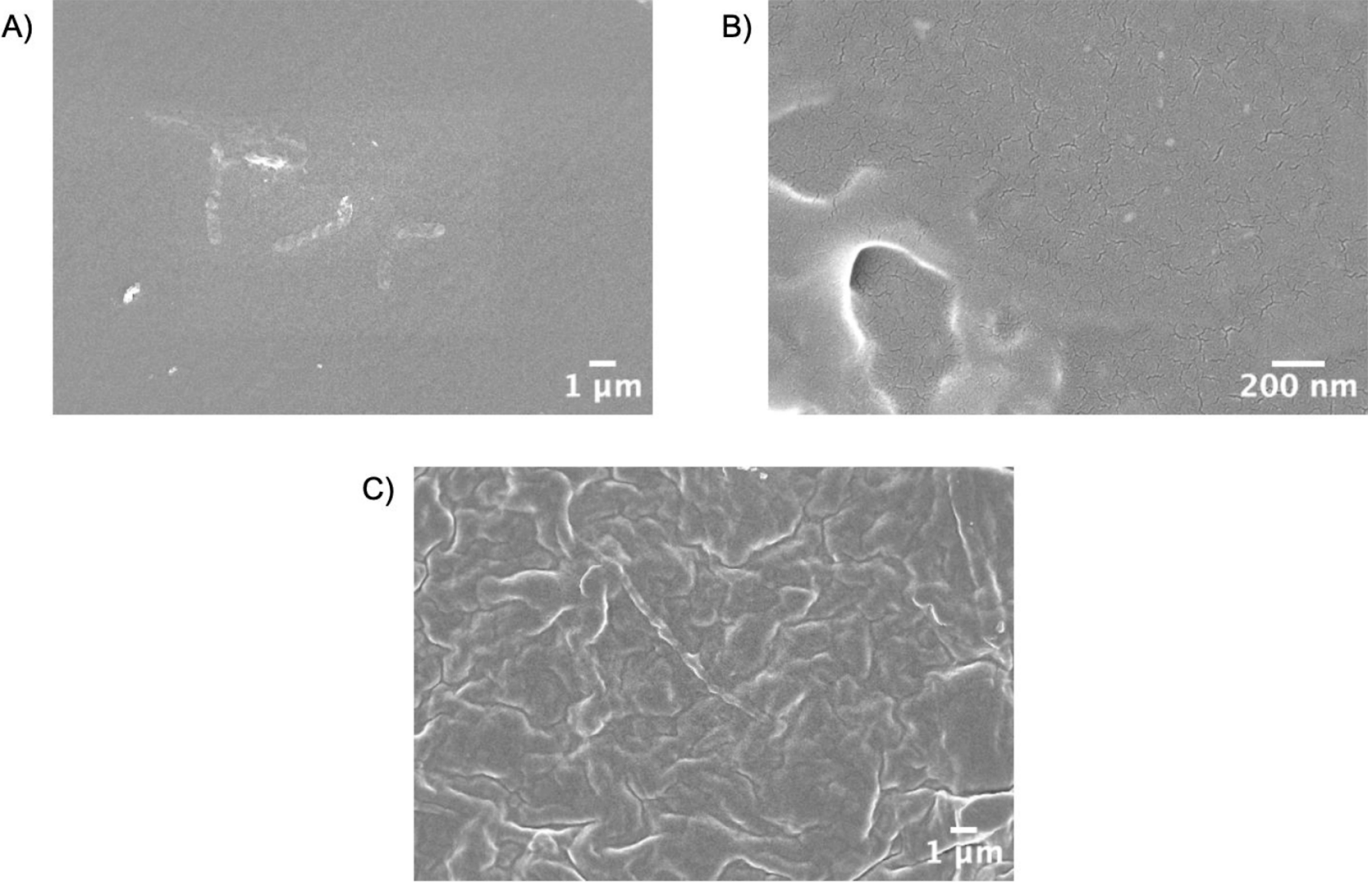
SEM images of (A) unmodified contact lens, (B) anthocyanin-stained contact lens, and (C) fCLs at pH 7.5.

In terms of the further characterization, FTIR spectra of CNF, MFX, unmodified lens, MFX-loaded lens, and CNF/MFX-loaded lens (fCL) are presented in Figure 5. The −OH and C–H stretching bonds in the CNF structure were attributed to 3248 cm^–1^ and 2883 cm^–1^, respectively (Figure 5A). The MFX gave weak and broad stretching vibration peaks of C–H from CH_2_ and N–H bonds at around 1445 cm^–1^ and 2900 cm^–1^. The unmodified contact lens led to stretching vibration peaks −OH, CH_2_, and −CH_3_ and C=O and C=C bonds at 3535 cm^–1^, 2958 cm^–1^, and 1721 cm^–1^, respectively, owing to the silicone structure (Figure 5C). All these peaks mentioned above in unmodified contact lenses appeared in all spectra of modified lenses. Figure 5D,E presented stretching vibration peaks of −OH, aliphatic carbon bonds, C=O, C=C, and N–H bonds, respectively, at around 3500 cm^–1^, 2958 cm^–1^, and 1720 cm^–1^.

**Figure 5:**
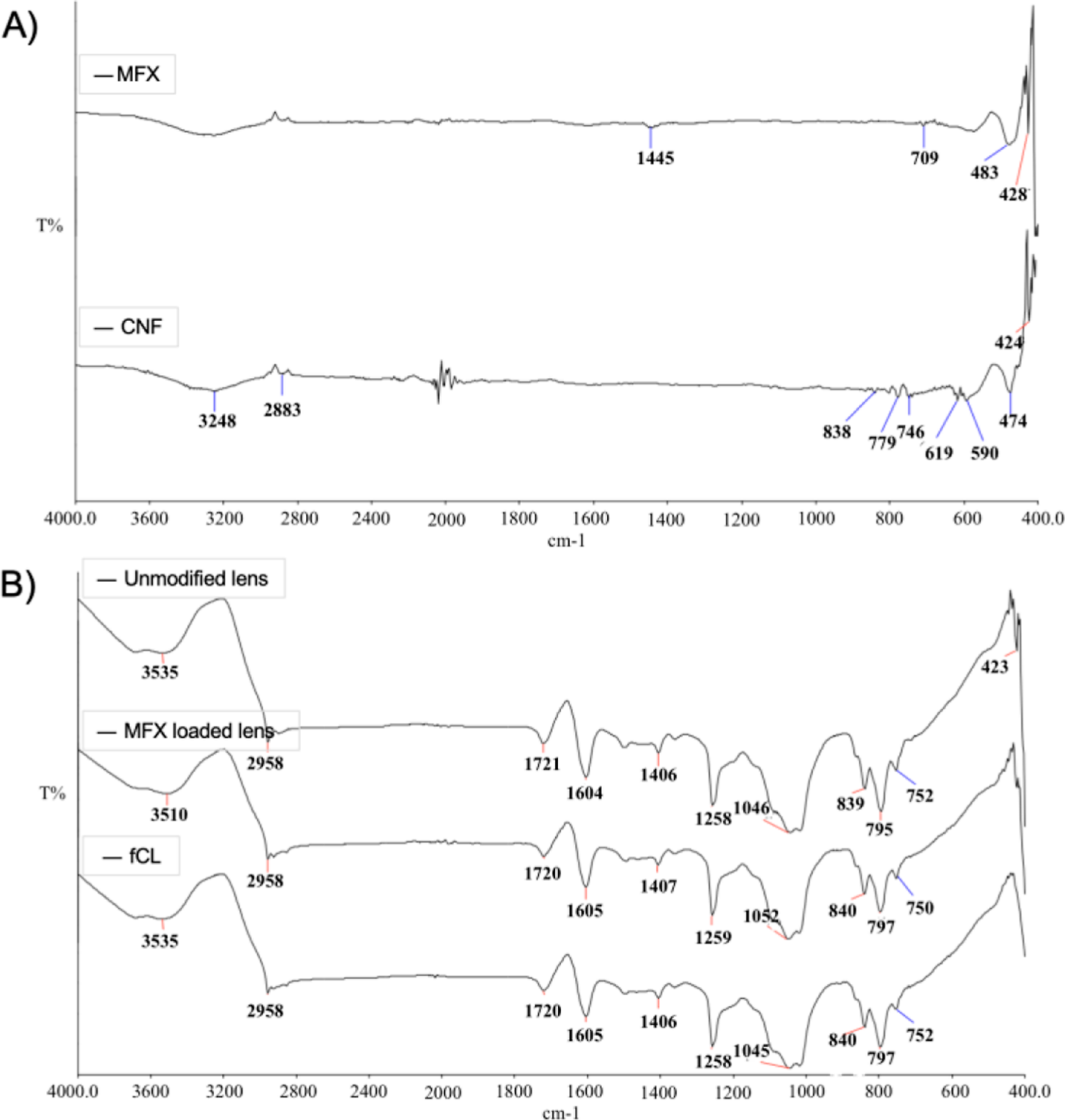
FTIR spectrum of (A) CNF and MFX, (B) unmodified lens, MFX-loaded lens, and functionalized contact lens (fCL) at pH 7.5.

High lipophilicity facilitates penetration of MFX into the CNF and corneal epithelium.^38^ *In vitro* drug release profiling of the MFX-loaded and the fCLs was performed. The concentration of MFX in the media was determined using a UV–visible spectrophotometer. For 12 h, drug release from the MFX–CNF nanocomposite occurred. This suggests that the efficacy can be improved by reducing the frequency of administration compared with the current dose frequency for the treatment of bacterial infection.

The drug release profiles of fCLs without CNF and fCLs were evaluated based on cumulative concentration (%) and are shown in Figure 6. fCLs prepared without CNF at pH 6.5, 7–7.5, and 8 released over 50% of the MFX in their structure within 6 h, whereas fCLs at the same pH values exhibited a release profile of 50% or less of MFX. Instead of rapidly releasing MFX over a 6 h period, the fCLs exhibited a controlled and sustained release profile. Particularly, at pH 6.5, which simulates an infection, the cumulative release of MFX from the fCLs was quantitatively lower (Figure 6A). We can attribute this situation to the CNF structure, which effectively enhances the MFX binding efficiency and inhibits its rapid diffusion. The presence of hydroxyl (−OH) groups in the CNF structure facilitates strong molecular interactions, thereby influencing both lens binding and MFX binding efficiencies.^39^

**Figure 6:**
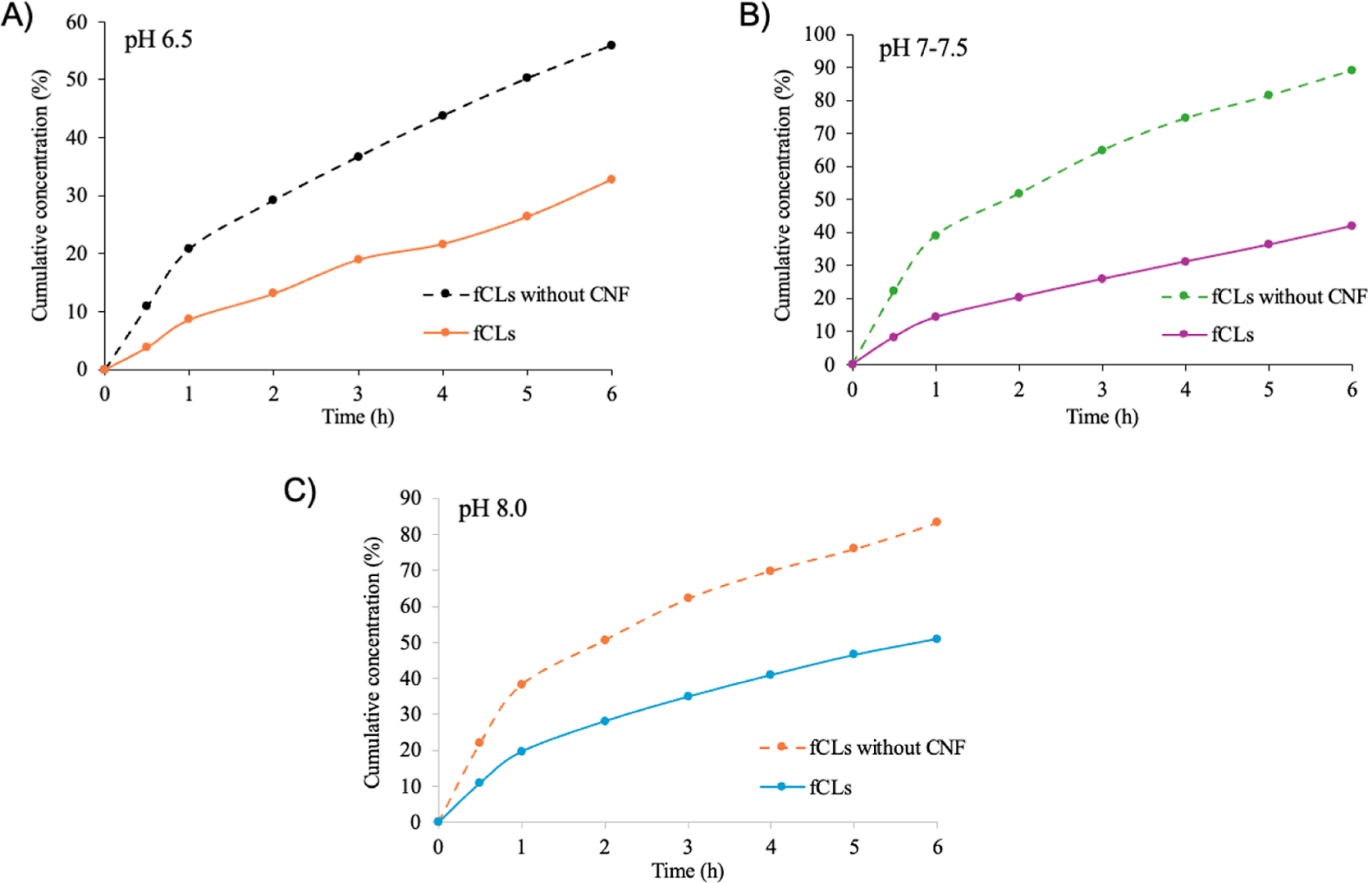
Cumulative drug release (%) profiles of MFX-loaded and CNF/MFX-functionalized contact lenses (fCLs) at (a) pH 6.5, (b) pH 7.5, and (c) pH 8.0.

CTAB was incorporated with pH indicator mixtures to prevent leakage of pH-responsive dyes, which can have detrimental effects, as previously reported,^40^ and altering p*K*_a_ of the dyes resulted in highly sensitive color changes according to pHs.^41^ In the infectious state, acidic shifts in tear pH of approximately from 7.4 to 6.5 have been reported.^42–44^ When we analyze the *in vitro* drug release profiling of the fCLs without CNF and fCLs in PBS buffer solutions with various pHs (pH 8.0, 7.5, and 6.5) by using a UV–Visible spectrophotometer. The MFX was rapidly released from the surface of fCLs without CNF at all three pHs compared to fCLs. The MFX release speed and ratio were affected by pHs. For instance, much MFX was rapidly released from fCLs without CNF at pH 8 (Figure 7A) and pH 7.5 (Figure 7B) compared to pH 6.5 (Figure 7C) in the first 0.5 h. The increase in absorbance value of MFX is proportional to the amount of released MFX. In terms of CNF use, the release profile of MFX is quite low from the surface of fCLs, which represents that CNF in fCLs provides less and controlled release owing to interaction between MFX and CNF. MFX release was much more rapid at pH 8 (Figure 7D) and pH 7.5 (Figure 7E) compared to pH 6.5 (Figure 7F) in the first 0.5 h. At ocular pH, MFX exhibits a zwitterionic and soluble form, a behavior that increases its diffusion in the hydrated lens matrix.^45^ On the other hand, in high pH environments (especially pH 8.0), MFX deprotonates, and the CNF surface is affected by electrostatic interactions.^46^ We interpret that MFX weakly binds to the surface of fCLs without CNF, and then much more rapid MFX release was observed compared to the release rate of MFX from fCLs. The low and controlled release of MFX at pH 6.5 can be a desired situation because the pH of an infected eye becomes acidic, typically between 6.5 and 7.0. The long-lasting time of MFX release may protect eye health compared to high-releasing behavior and much MFX cumulative concentration.

**Figure 7:**
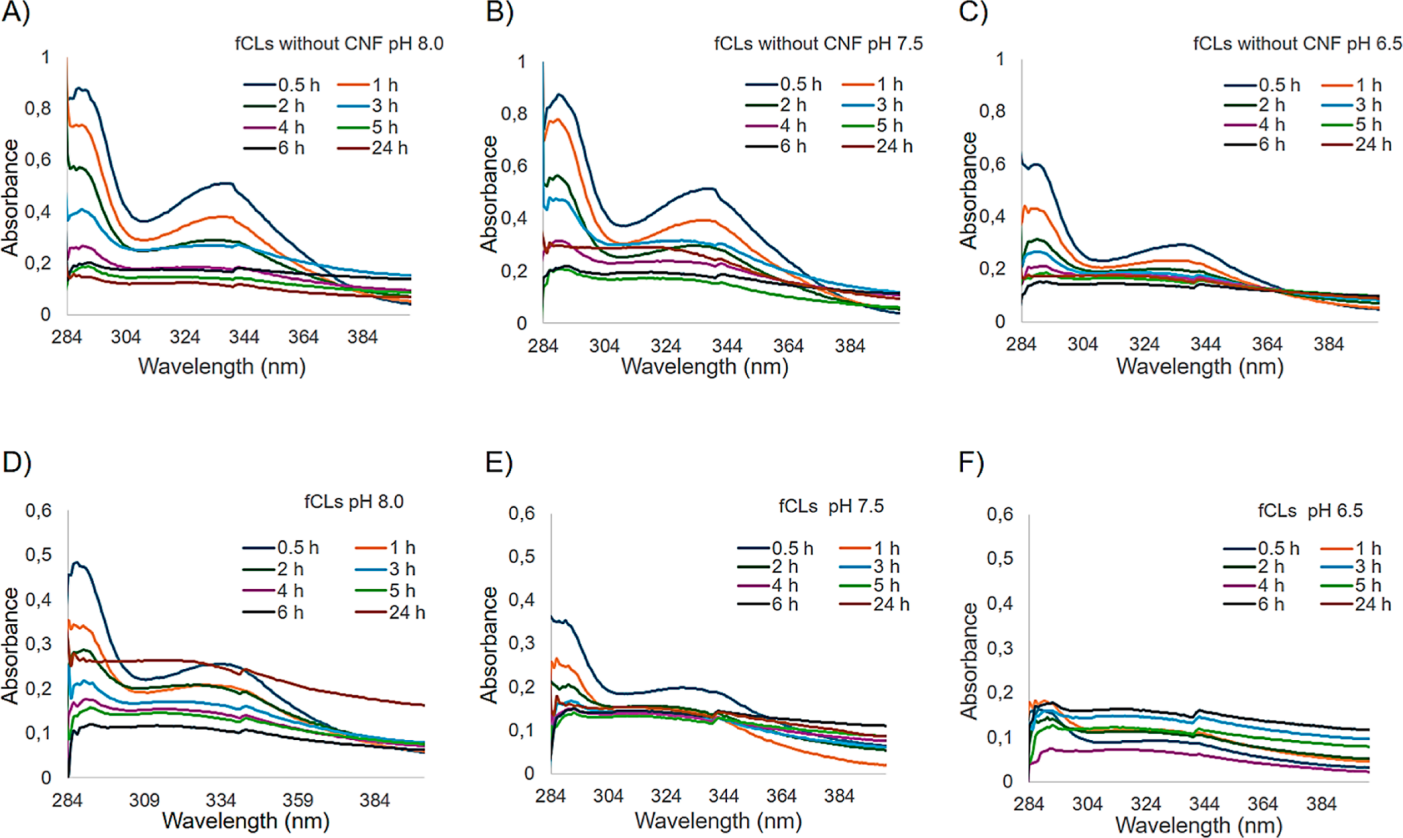
UV–visible spectra of MFX released from (A) fCLs without CNF and (B) fCLs at different pHs.

We examine the antimicrobial activities of fCLs without CNF and fCLs toward *E. coli*, *S. aureus*, and *E. faecalis*. The fCLs without CNF and fCLs were soaked into PBS solutions at different pHs (pH 8.0, 7.5, and 6.5). The MFX was released from fCLs without CNF and fCLs in various incubation times, including 30 min, 1, 2, 3, 4, 5, 6, and 24 h. Then, each supernatant collected from different incubations containing released MFX was incubated with each bacterial suspension for a different period of time. For instance, the MFX released from fCLs without CNF at pH 8.0, 7.5, and 6.5 was mixed with *E. coli*, *S. aureus*, and *E. faecalis* suspension, and then each mixture was incubated for 24 h (Figure 8A).

**Figure 8:**
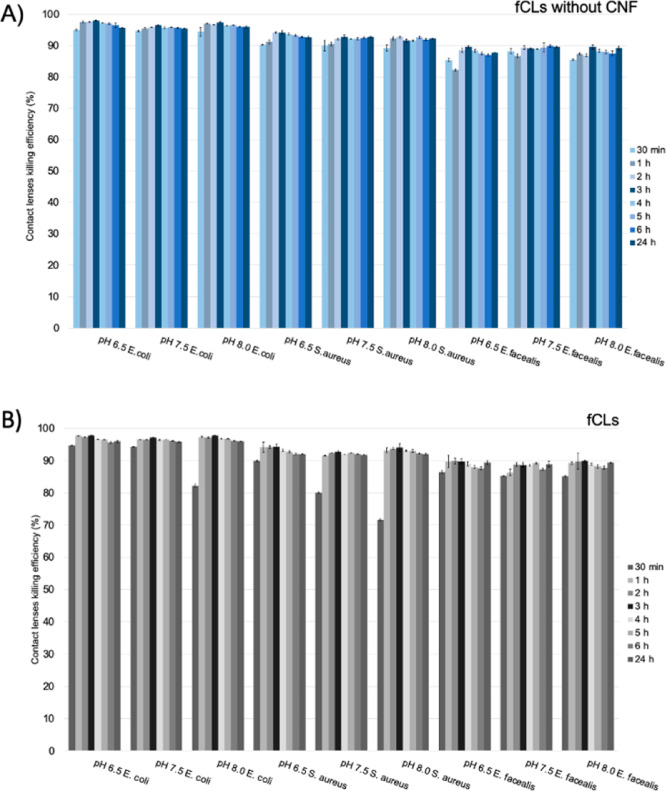
Killing efficiency of MFX released in different incubation times and at different pHs from the surface of (A) fCLs without CNF and (B) fCLs.

In fCLs without CNF, the inactivation of over ∼90% of bacteria was observed across all strains and pH values during the 30 min to 24 h incubation period (Figure 8A). The limited binding of MFX to the surface of fCLs without CNF resulted in rapid burst release, leading to enhanced bactericidal activity within a short incubation period. In comparison with fCLs without CNF, fCLs demonstrated ∼70% and above cell inactivation in *E. coli* cells during the initial 30 min incubation period. In the incubation period range of 1–24 h, an increase of ∼90% and more was observed in the antibacterial activity of fCLs. In this context, it was observed that the release of MFX from the surface of fCLs was rapid in the first stage and sustained its high bactericidal activity with the continuation of release in the following period (Figure 8B). Accordingly, CNF modification to the lens surface enabled the controlled release of MFX and resulted in marked bactericidal activity against common ocular pathogens such as *E. coli*, *S. aureus*, and *E. faecalis* *.*

The antibiofilm activity of fCLs without CNF and fCL was evaluated by SEM imaging against *E. coli* biofilm formation under different pH conditions (8, 7.5, and 6.5). The analysis showed significant differences between fCLs without CNFs and fCLs in the tested medium. The intensity of biofilm formation exhibited differences, depending on the pH value in the lenses. However, the biofilm was consistently observed at high density in fCLs without CNF (Figure 9A). In this regard, a relatively reduced biofilm coating was observed on the surface of fCLs without CNF at pH 6.5 (Figure 9A­(iii)), while intensive biofilm layers were clearly visible at pH 8 and 7.5 (Figure 9A­(i),(ii)).

**Figure 9:**
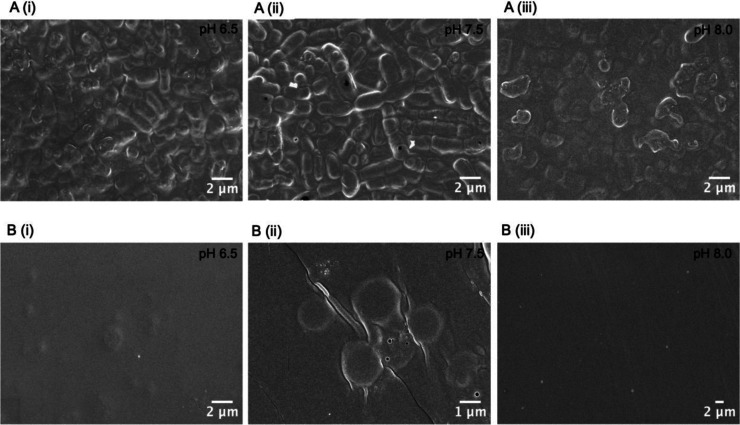
SEM images of (A) fCLs without CNF and (B) fCLs at different pHs and their antibiofilm activity against *E. coli*.

As shown in Figure 9B, fCLs exhibited significant antibiofilm activity at different pH conditions (8, 7.5, and 6.5). Minimal bacterial residue was observed at pH 7.5 (Figure 9B­(ii)), while at pH 6.5 and 8, the surfaces of fCLs exhibited an almost completely clean surface (Figure 9B­(iii),(i)). In antibiofilm efficacy tests against *E. coli*, CNFs provided effective penetration and controlled release of MFX from the fCLs surface. Under physiological pH conditions (6.5–7.5), fCLs exhibited strong antibiofilm activity and prevented *E. coli* colonization on the lens surface.

## 4 Conclusion

We successfully developed fCLs by integrating anthocyanin-based natural and biocompatible pH indicators with MFX-loaded CNFs on the lens surface. Anthocyanin functionalization of the lens surface provided a rapid, visible, and sensitive colorimetric response to ocular pH changes related to infection. Compared with fCLs without CNF, the CNF content of fCLs enhanced the effective penetration of MFX into the lens surface and its sustained release from the surface. The fCLs exhibited potent antibacterial activity against common ocular pathogens such as *E. coli*, *S. aureus*, and *E. faecalis*, providing significant antibiofilm protection against *E. coli*; protective and bactericidal efficacy was sustained at physiological pH values (6.5, 7.5, and 8.0). Furthermore, we observed that fCLs exhibit a strong bactericidal performance, reducing bacterial colonization on the lens surface. The developed fCLs combine antibacterial and antibiofilm properties with pH-responsive sensing capability, demonstrating potential as multifunctional ocular devices for monitoring and treating ocular infections. However, it should be noted that these findings are based on in vitro studies, and further in vivo and clinical investigations are required to validate their practical applicability.
